# Impacts of Human Robot Proxemics on Human Concentration-Training Games with Humanoid Robots

**DOI:** 10.3390/healthcare9070894

**Published:** 2021-07-15

**Authors:** Li Liu, Yangguang Liu, Xiao-Zhi Gao

**Affiliations:** 1 College of Digital Technology and Engineering, Ningbo University of Finance and Economics, Ningbo 315175, China; liuli6883@nbufe.edu.cn; 2 College of Finance and Information, Ningbo University of Finance and Economics, Ningbo 315175, China; 3 School of Computing, University of Eastern Finland, 70210 Kuopio, Finland; xiao-zhi.gao@uef.fi

**Keywords:** human robot proxemics, human robot interaction, concentration training, psychology response, proxemic distance, nonverbal behavior

## Abstract

The use of humanoid robots within a therapeutic role, that is, helping individuals with social disorders, is an emerging field, but it remains unexplored in terms of concentration training. To seamlessly integrate humanoid robots into concentration games, an investigation into the impacts of human robot interactive proxemics on concentration-training games is particularly important. In the case of an epidemic diffusion especially—for example, during the COVID-19 pandemic—HRI games may help in the therapeutic phase, significantly reducing the risk of contagion. In this paper, concentration games were designed by action imitation involving 120 participants to verify the hypothesis. Action-imitation accuracy, the assessment of emotional expression, and a questionnaire were compared with analysis of variance (ANOVA). Experimental results showed that a 2 m distance and left-front orientation for a human and a robot are optimal for human robot interactive concentration training. In addition, females worked better than males did in HRI imitation games. This work supports some valuable suggestions for the development of HRI concentration-training technology, involving the designs of friendlier and more useful robots, and HRI game scenarios.

## 1. Introduction

Thanks to the advances in robotic technology, human robot interaction (HRI) is popularly used for a variety of applications, including in-store sales [1], entertainment [2], education [3], personal healthcare [4], and therapy [5]. As an increasing amount of the human workforce is replaced by robots, HRI should naturally be widely used in education [2,4]. The application of HRI in education faces many challenges [5], such as how people accept robot partners, how human robot interactive proxemics influence the experiences of humans, and how robots work with humans, such as human co-workers, especially in special-education areas. Human concentration training is an important skill in special education [6]. Imitation learning is an important, widely used method in concentration training, by which an agent tries to mimic an action performed by another [7]. There are four crucial indicators for the assessment of concentration—namely, imitation accuracy, short-term memory, attention stability, and persistence [8,9,10]. This provides a powerful mechanism whereby knowledge may be transferred between agents (both biological and artificial).

### 1.1. Imitation Learning

A significant number of studies have been published on imitation learning in animals and humans that state that imitation should be triggered by mirror neurons that are active both during action execution and during perception of one’s learning partner performing the same action [7]. They proposed that familiar environments are conducive to stimuli, and imitating should trigger a familiar or unfamiliar response in how a stimulus changes. Stéphane and co-workers found that many sulcus neurons are excited by the actions of specific body parts of an observed human, which seem to be the perfect candidates for the first processing step of imitation [8,9,10]. Butler indicated neurons in area F5 (a cortical area that contains neurons endowed with mirror properties) that are sensitive to the performance of goal-related actions, e.g., “pushing”, “leg lift”, and “handshake”, and suggested that action imitation can promote the development of social skills [11]. Maurtua and co-workers indicated that humanoid robots can compellingly and autonomously play with humans in educational games, replacing the human teacher during the process [12]. Therefore, action imitation is an excellent candidate for human concentration training. However, imitation is impacted by whether agents belong to the same social group, and by whether the context is competitive cooperation [13,14]. The aim of these previous investigations in HRI was to investigate how humans and robots interact together in a shared physical space while accomplishing a goal [15]. Thus, a human cognizes a robot partner in HRI imitation depending on the physical interaction, distance, actions, and the environment itself.

### 1.2. HRI Imitation

The crucial consideration for HRI imitation is proxemics, which typically contains the physical (e.g., physical distance and orientation) [16] and psychological (e.g., mutual gaze or willingness) expressions [17] of an interaction. Humans may recognize robots that have no suitable distancing behavior as a threat and obstruction to their social work. Physiological affection is also a crucial factor in HRI games because it directly impacts the willingness of humans to accept robot-executed information, following robot representation [18]. The recognition of emotional expressions and the perception of emotions in general plays a crucial role in social interpersonal communication [19]. Wainer provided a probabilistic framework for psychophysical expression to bridge the gap between these physical and psychological expressions by considering the cognitive experience of each agent in HRI. Robots with appropriately proxemic behaviors might obtain human acceptance well, contributing to their seamless integration into various applications [20]. Jerčić and Lindley suggested that serious games which are carefully designed to take into consideration the elicited physiological arousal might witness better decision-making performance and more positive valence using nonhumanoid-robot partners instead of human ones [21]. Liu showed that embodied nonhumanoid robots are as engaging as humans, eliciting physiological arousal in their human partners [22]. Evidence further indicates that human are sensitive to the environmental cues of cooperative robots, which easily elicits the physiological affection of human partners [23,24]. To the best of our knowledge, there are very few studies on HRI imitation games for human concentration training, and no guidelines exist for the future design of proximity behaviors for robots in concentration training [25,26]. For example, it would be undesirable if human robot proxemics in the HRI games were not suitable, because such behavior comes across as unintelligent and unfriendly [27]. Hence, researchers need to know whether people are likely to assess the distance between the robot and human when they observe them, and which factors can modulate those perceptions [28].

## 2. Materials

### 2.1. Human Robot Interactive Game

Current methods to investigate HRI games fall into two categories: behavioral and psychological approaches. For behavioral research, because games are played covering a variety of activities, no precise definition of gameplay has been presented [29]. Many methods deal with gameplay and research this field differently in terms of their special purposes. Games may exhibit two different representations: active and passive learning. All forms of gameplay need human interest, concentration, and mental activity [30]. Psychological research on HRI games involves many factors, such as preferences, comfort, security, and happiness. Some research related to HRI games was performed, but the studies mainly focused on the relationships between people and their robot players [31]. There have been some studies on the effects in collaborative HRI games, and the design of a context-aware proxemic planner which aims to improve a robot’s social behavior by adapting its distance management [32].

### 2.2. Human Robot Proxemics

**Figure 1:**
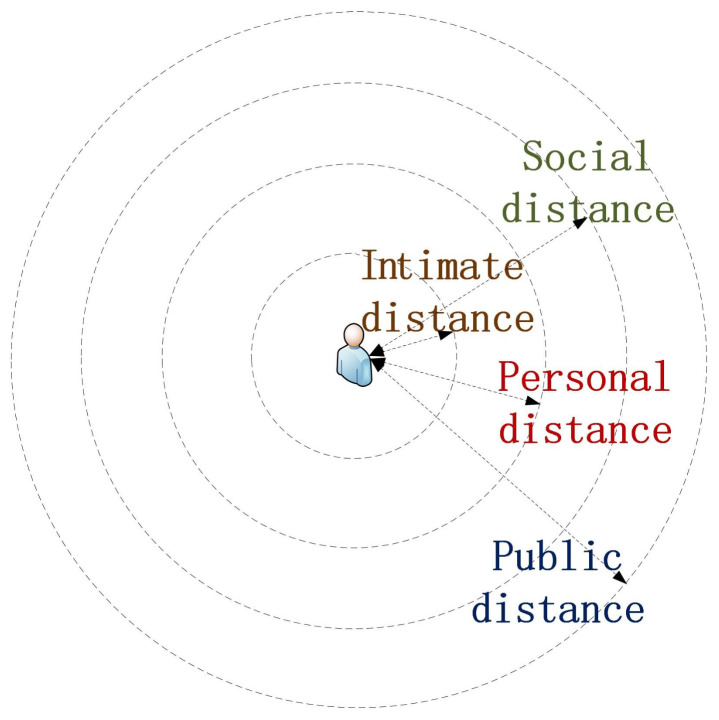
Relationships between interpersonal position and sensory experiences.

Impact factors of HRI concentration-training games usually contain human robot interactive distance, proxemic direction, robot size and appearance, and the environment [27,28,29,33,34,35]. The first two factors significantly influence people’s experiences with and perceptions of a human-like robot in HRI games [6]. Physical interpersonal distances should conform to societal norms (relative distances between people) that are expressed in four distinct zones, i.e., intimate space, personal space, social space, and public space, as shown in Figure 1 [36]. The space between intimate and personal distance is called personal space (ranging from 0.46 to 1.22 m). The space between social and personal distance is called the social space (ranging from 1.2 to 3.7 m). The space within public distance is called the public space (ranging from 3.7 m to infinite).

Human proxemic behavior contains physical and psychological distance. There are some papers related to interpersonal distances [37] and the fixed distances among human groups [38].

### 2.3. Human Concentration Training

Concentration is essential for humans. It is giving attention to a task, which is good for performing at one’s best while not being affected by irrelevant external and internal stimuli [39,40,41,42]. External stimuli involve the external environment, context, and voices. Concentration or attention is very important in sport psychology [43]. It is evidently difficult to study the processes of some people because of the lack of concentration [44]. The use of robots in the concentration-training context offers students new effective learning strategies in HRI spaces through a personalized and unique experience. With suitable interaction schemes, the usage of HRI concentration-training games could improve participant performance [45].

### 2.4. Hypotheses

Some promising studies in human robot interaction have explored proxemic behavior, as described in the last section. These studies show promising evidence that people express proxemic preferences when they are interacting with robots [2,29,30,44], but comprehensive theoretical models or experimental results of physical and psychological distancing are needed to guide the design of proxemic behaviors for robots. We formed three hypotheses for human robot proxemics in concentration-training games based on the models that presented findings from human robot interaction studies [27,36,37,46].

**Hypothesis** **1.** *Following perceptual models of human robot proxemics [44], outcomes are derived from nonverbal behaviors, which explains the impacts of human proxemics on the effectiveness of HRI, assuming that the physical distance between human and robot is face-to-face during HRI imitation play.*

**Hypothesis** **2.** *Following human proxemics [46], to understand how people physically and psychologically relate to robots compared to other humans, direction has little effect on HRI concentration-training games. Therefore, direction has little impact on the accuracy rate of action imitation, and the right-front direction has a slightly larger effect for face-to-face HRI games.*

**Hypothesis** **3.** *Following existing studies of human proxemics, the best HRI distance for face-to-face, front-on imitation games is thought to be 1–2 m, and the effectiveness of HRI imitation games, e.g., comfortability and fun, is significantly impacted.*

In the next section, a controlled laboratory experiment is described in which these hypotheses were evaluated in a human robot interaction scenario.

## 3. Methods

A controlled laboratory experiment was designed to explore how human robot proxemics influence HRI concentration training by action-imitation games in which a tester demonstrates random movements, and participants are to immediately repeating them (approximately). Experimental datasets, the procedure, measurements, results, and participant information are described below.

### 3.1. Experimental Conditions

**Figure 2:**
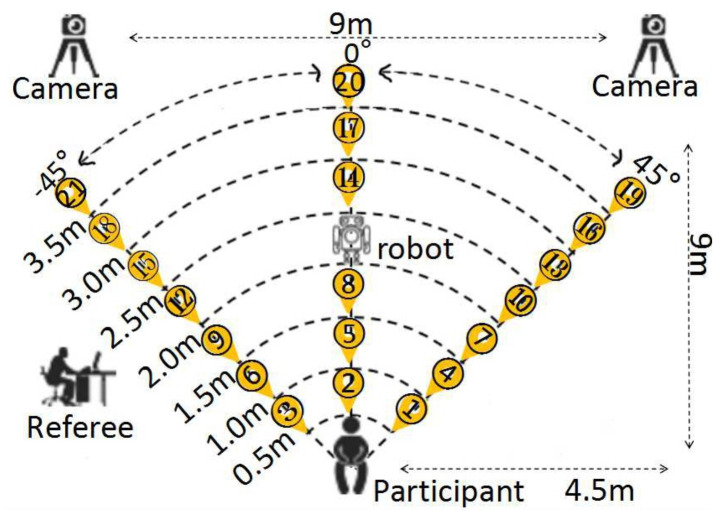
Experimental setup of concentration-training games with a humanoid robot.

The experiments consisted of a game scenario involving a participant, a tester, and an operator. The tester could be either a human or a semiautonomous robot that was manipulated by the operator. The controlled-play scenario was in an enclosed laboratory with controlled light that was free from outside distractions. The width of the experimental site was 11 m, and the length was 13 m. During the game, the participant sat on a chair against the wall facing the tester, who could not stand up or turn. The tester was fixed face to face with the participant, and the directions in front of the participant were set from left to right as $-45^{\circ },0^{\circ }$, and $45^{\circ }$. The distance between participant and tester was divided into seven different steps (from 0.5 to 3.5 m with a step of 0.5 m) and three different spatial directions. There were 21 position tags set on the floor by distance and direction between participant and tester that were numbered from 1 to 21, as shown in Figure 2.

The experimental equipment was one laptop, one humanoid robot, two cameras, one chair, and one game positioning tag. The humanoid robot was controlled to move semiautonomously by an operator, executing nonverbal action like a human. The testers were a human tester and a humanoid-robot tester.

### 3.2. Participation

The participants were 120 students with an age range from 17 to 20 invited by a local university: 60 females and 60 males. All students could perform normal imitation behaviors according to the testers; they had no difficulty in movement and were accepting of the game.

### 3.3. Experimental Design

In our experiments, every participant would play random action-imitation games with a tester to evaluate concentration. The imitation games comprised two modules for every participant, namely, playing with the human tester and with the humanoid-robot tester. Researchers conducted two modules of imitation games for every participant at every experimental position (from 1 to 21), which alternately started with the human or robot player. Each participant needed to successively perform three random continuous actions mimicking the tester, including left or right-leg lifting, left or right-hand raising, and raising both hands. After the tester finished executing an action, the participant had to mimic the action for no more than 3 s.

A points system was utilized to judge whether the participant would win the game, and the rules of the game were as follows. One point was awarded if the participant accurately mimicked the action within the specified time; otherwise, no point was awarded. The maximal score for one participant was 84 points. If the participant got 76 points or more, they won the game. At the end of the game, each participant was asked to complete a questionnaire containing eight open-ended questions. Each question was graded on a scale of 1 to 5, representing “strongly dislike” to “strongly like.” After answering the questionnaire, the game ended, and the next participant played the game [47].

### 3.4. Experimental Procedure

Only a tester, a participant, and a referee were present for the game. When the experiment started, the participant was asked to sit down and direct their concentration to the operator, who introduced the rules of the human–human interactive (HHI) concentration game [31,36]. When the operator finished the introduction of the game, they confirmed that the participant had clearly understood the rules of the game. Then, the participant began to play the imitation game.

Every participant played with a human tester and a robot tester. In order to achieve the objective and reasonable experimental results, every participant played with the same tester for 2 rounds with a sequence of (1,2,3,⋯,21) and an opposite sequence of (21,20,19,⋯,1). Random actions were determined by the tester regardless of sequence. Random imitation games mainly related to the choice of body posture and not the sequence.

### 3.5. Measurement

There were three independent manipulated variables in our experiments: (1) humanoid-robot size, (2) humanoid-robot appearance, and (3) random actions of the tester. All independent variables were operated by the tester. The dependent variables involved in the participant measurements related to imitation accuracy, comfortability, and fun were proxemic distance and direction. The imitation games with the human tester were compared to those with the robot tester by using the combination methods of imitation accuracy, assessment of emotional expression, and questionnaires. The impacts of distance and direction on the imitation games were explored, thereby finding the optimal human robot proxemics for HRI imitation games.

## 4. Results

Analysis of the experimental results was related to the physical distance between and orientations of participants and the tester using analysis of variance (ANOVA) [37,39]. All experimental results were processed and analyzed by SPSS software. Analysis of imitation accuracy was mixed-effects repeated measures ANOVA, where physical distance and direction were random effects, but imitation actions and robot appearance conditions were fixed effects. The two other independent variables, participant gender and age, were fixed effects. Psychological distance was analyzed using the questionnaire method.

### 4.1. Proxemic Distance and Direction

**Figure 3:**
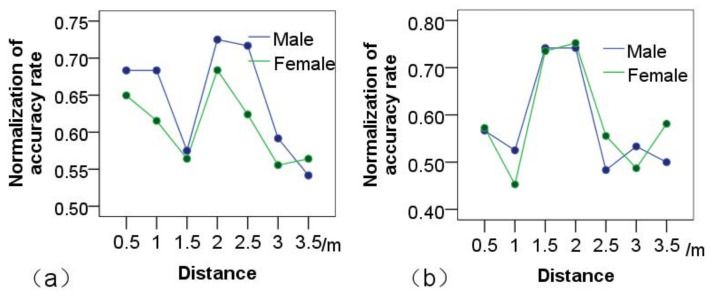
Comparison of the normalization of accuracy rate at different distances for action imitation. (**a**) HHI games; (**b**) HRI games.

Physical distance: experimental results demonstrated the main effect of physical distance on the imitation-accuracy rate of HHI or HRI games. The proximity distance between participant and tester significantly influenced HHI imitation games, F(1,6)=3.35,p<0.01, as shown in Figure 3a.

At seven different orientations at 2 m, the highest imitation-accuracy rate was achieved, F(13,804)=2.98,p<0.01. Beyond 3 m, the accuracy rate was linearly decreased. At the same time, the proximity’s influence on imitation-accuracy rate was analyzed. Analysis proved that proximity significantly influenced the accuracy rate of the HRI imitation game: F(1,6)=12.52,p<0.001, as shown in Figure 3b.

As in the HHI game, the highest accuracy rate of the imitation game was at the physical distance of 2 m, F(13,804)=3.484,p<0.001. The distance between the participant and the human or robot tester therefore had an obviously significant influence on the concentration-training game. Therefore, the experimental results confirmed the hypothesis that, at 2 m, participants have the best imitation accuracy. This was the case for both HHI and HRI imitation games, as shown in Figure 3.

**Figure 4:**
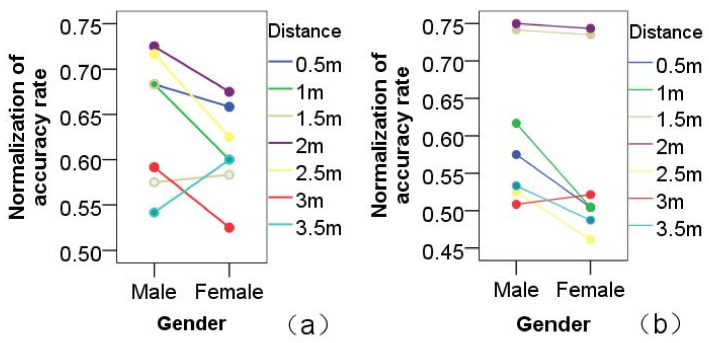
Distance analysis: effect of distance on action-imitation games played by participants of different genders in different game scenarios. (**a**) HHI games; (**b**) HRI games.

Furthermore, the influence of gender on the concentration-training game was also analyzed. In the HHI game, experimental results demonstrated that male participants had slightly higher accuracy than female participants. The influence of gender on the accuracy of the concentration games was small: F(1,804)=1.239,p>0.05, as shown in Figure 4a. In HRI games, results demonstrated no significant difference between males and females: F(1,804)=0.077,p>0.05, as shown in Figure 4b. At 2 m distance, male and female participants were almost equally accurate.

**Figure 5:**
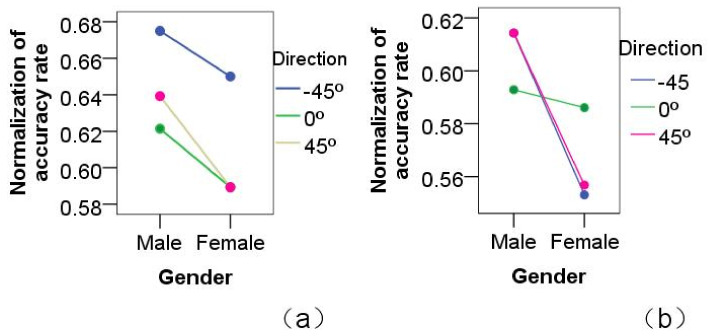
Direction analysis: the effects on action-imitation games of gender, different directions, and different game scenarios. (**a**) HHI games; (**b**) HRI games.

Proxemics direction: results showed that direction is another significant factor that influences concentration games. In HHI games, direction was the main impact factor. Results showed that there were different accuracy levels when the tester was in different directions: F(2,2457)=2.899,p<0.05. The accuracy of the HHI games was higher when the tester was at $-45^{\circ }$, rather than at other directions: F(2,360)=2.589,p<0.05, as shown in Figure 5a. In HRI games, results showed that direction was an impact factor, but not significantly: F(2,2425)=1.699,p>0.05, as shown in Figure 5b. According to analysis, the HRI game’s results were similar for $-45^{\circ }$ and $45^{\circ }$. At the same time, males had an obviously better accuracy rate than that of females for any direction, especially in HRI games. Analysis confirmed Hypothesis 1, and the direction of $-45^{\circ }$ was more conducive to the face-to-face HRI game.

**Figure 6:**
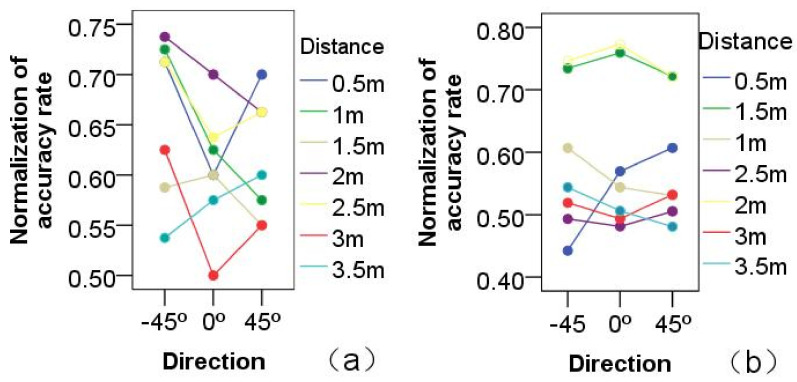
The effects of different proxemic distances and different directions. (**a**) HHI games; (**b**) HRI games.

Additionally, comparative results of the influences of direction on HHI and HRI imitation games are shown in Figure 6a,b, respectively. By comparatively analyzing the experimental results of the two different modules of imitation games, the impact of direction on HRI games was shown to be less than on HHI games. Experimental results showed that Hypothesis 2 was valid.

### 4.2. Perception of Students’ Emotional Expression

Researchers discussed students’ imitation accuracy in the interaction games with humans, and compared the results with those obtained by students playing with the humanoid robot. During the whole experimental procedure, the participants were videotaped. There were various types of nonverbal social behaviors and emotional responses to winning or losing in a game. In this section, we analyze the emotional responses from selected recordings of the participants that were taken under HHI or HRI conditions by third-party judges. A judge’s task was to evaluate via the video clips whether a participant had won or lost the game. By this method, the expressiveness of the participant would objectively be estimated in different experimental conditions, and indicate whether participants were more expressive via a more correct estimation.

Forty student observers were invited to judge whether participants won or lost games by observing their emotions in the video clips. The student observers were divided into four groups. Each group was invited into a classroom where the representative frames from video clips were projected onto a wall. Six different frames were shown in order at a time. In 5 s, observers had to make a judgment and write the score on a piece of paper.

**Figure 7:**
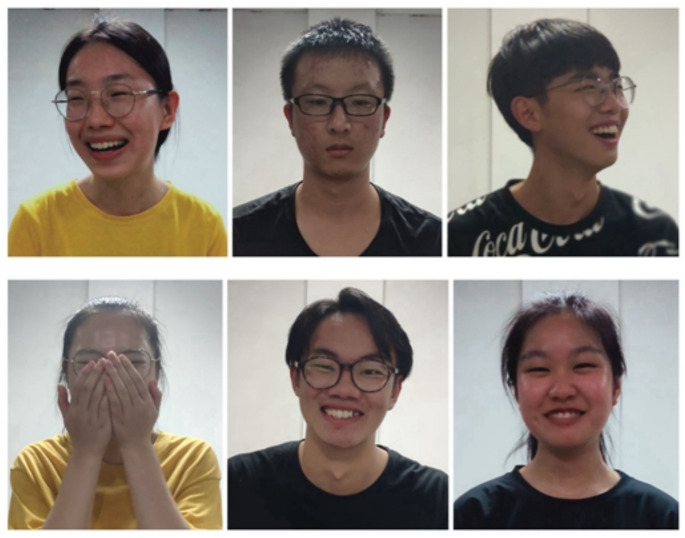
Representative stills of undergraduates’ reactions after winning or losing a game while playing with a human (**top**) or robot (**bottom**).

The researchers analyzed the data from the two different scenarios to study significant effects for the concentration games by comparing judgment accuracy. For collected data in various experiments, the two main scenarios of interest (human and robot testers) were statistically compared with independent-sample t-tests. The judgmental-accuracy rate of the observers for the HHI game (M = 0.87) was slightly higher than that for the HRI game (M = 0.80), t (553) = 5.01, *p* < 0.001. Therefore, participants were more expressive in HHI concentration games than in HRI games. The expressions of female and male students were compared. Male students (M = 0.90) were more expressive than female students (M = 0.81) in HHI games, as shown in Figure 7. However, in HRI games, the judgment accuracy of the male participants was similar to that of female participants. Results showed that male students playing with humans were more expressive than female students in the HHI imitation games. However, in HRI imitation games, the male students playing with humanoid robots were as expressive as female students.

In addition, the effect of proxemic distance on a participant’s expressions during the game was studied. The accuracy of judgments for participants’ 297 emotional expressions at different distances in HHI and HRI games are summarized in Table 1 and Table 2, respectively. Accuracy of judgment at the 2 m distance was higher ((M=0.35),F(1,6)=12.87,p>0.001) than that at other distances in the HHI games. Similarly to HHI games, the percentage of judgmental accuracy rate was higher ((M=0.39),F(1,6)=14.52,p>0.001) at the 2 m distance than that in other distances in the HRI games. The percentage of judgmental-accuracy rate demonstrates that the participants’ expressions at the 2 m distance in the HRI game were more obvious than that at the 2 m distance in the HHI game. Thus, the effect of proxemic distance on the emotion expression in the HRI games was more obvious than that in the HHI games. The effect of proxemic direction on participant expressions during the game was also studied. The accuracy of judgment showed that different directions had little effect on the expression effect. In the next section, the psychological response is analyzed by questionnaire.

**Figure 8:**
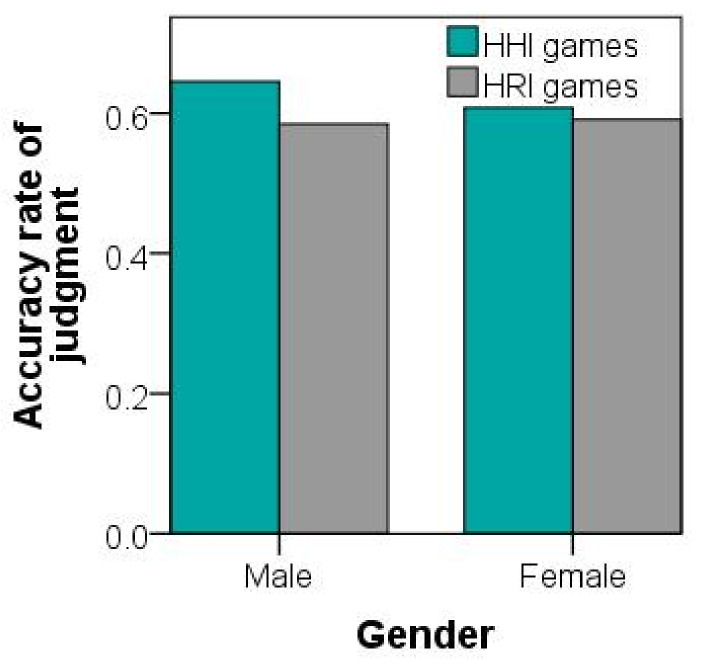
Accuracy rates of judgments for participants winning or losing by evaluating their emotional expressions in HHI and HRI games.

**Figure 9:**
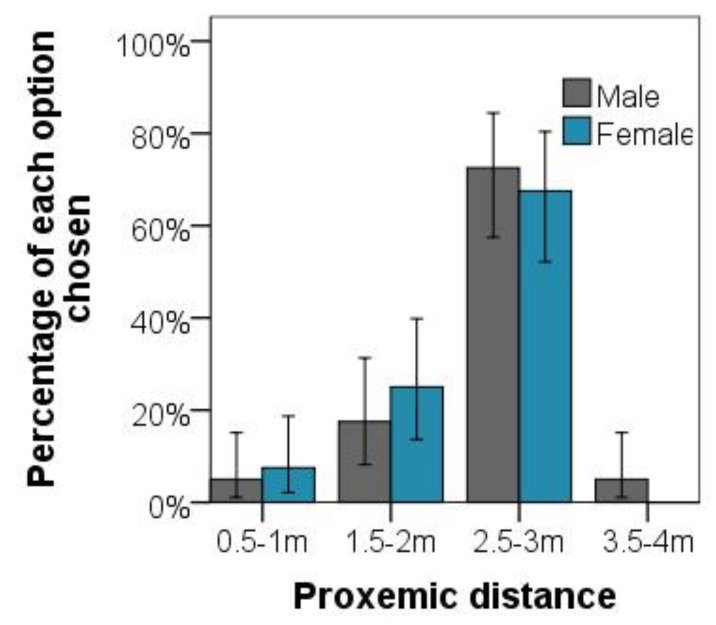
Presentation of selection results for each distance chosen by all student participants.

In the questionnaire investigation, our analysis showed that students preferred playing with humans (M = 0.56) over playing with robots (M=0.48),t(90)=8.01,p<0.001, as shown in Figure 8. Female participants disclosed a marginal preference for a human tester over a robot tester, F(1,129)=5.21,p<0.05. Our analysis further confirmed that proxemic distance had a more significant effect on participants’ play psychology in HRI games, F(1,6)=11.15,p<0.001 than that in the HHI games, F(1,6)=15.23,p<0.001. The range of 1.5 to 2 m distance was most people’s choice, as shown in Figure 9.

According to analysis, 2 m was the optimal proxemic distance in both HHI and HRI games. Analysis verified the hypothesis that 2 m distance was the best human robot distance for both HHI and HRI concentration-training games. Direction had little influence on the psychological experience in HRI games F(1,2)=2.05,p=ns, as in HHI games F(1,2)=1.12,p=ns. In summary, experimental results show that Hypothesis 3 is valid.

Various methods of analysis showed that the experimental results were continuous. The combined evidence of imitation accuracy, emotional-expression assessments and questionnaire investigation agreed with the hypothesis.

## 5. Conclusions

This paper provided a new approach to assess human concentration training by using an imitation game with a humanoid robot. The effects of proxemic distance and direction on the concentration-training game were analyzed with HHI and HRI imitation games. In total, 120 participants who were 18-year-old students from the same university were invited to play the imitation games.

On the basis of the findings, this study contributes to HRI research in the following ways.

- Direction for imitation is less important for robot trainers than for human trainers, so in a classroom, a robot may be placed at any angle in front of the learner.
- Suitable distance is good for trusting a robot, which is vital for subjects’ willingness to play with the robot.
- The different physiological effects in humans collaborating with a robot partner and a human partner were comparatively analyzed.
- Students of different genders responded to HRI and HHI games differently, which indicated that female students had more interest in playing the imitation game with a humanoid robot than male students did.
- Students felt that playing with people was similar to playing with humanoid robots.

To promote HRI instead of HHI games in human concentration training, future research should explicitly consider individual differences, such as cultural background and age, during the HRI game-design process. Humans are more interested in using HRI games because of an attractive robot implemented with smart objects. Overall, this study could inform the practice of HRI games, and the design of friendly and useful robots.

## Figures and Tables

**Table 1:** Numbers of occurrences of emotional expressions in HHI games.

Responsive Category	Emotional Expression	0.5 m	1 m	1.5 m	2 m	2.5 m	3 m	3.5 m
	Smile	200	147	88	94	126	111	134
Win	Laugh	41	79	116	139	108	86	77
	Winning gesture	2	5	5	9	8	5	0
	Total positive features	243	231	209	242	242	202	211
	Frown	114	86	87	51	63	87	96
Loss	Closing eyes	5	44	62	55	52	66	55
	Head down	0	0	2	1	2	4	2
	Total negative features	119	130	151	107	117	157	153

**Table 2:** Numbers of occurrences of emotional expressions in HRI games.

Responsive Category	Emotional Expression	0.5 m	1 m	1.5 m	2 m	2.5 m	3 m	3.5 m
	Smile	135	124	150	171	89	99	123
Win	Laugh	54	72	105	94	83	85	59
	Winning gesture	6	6	8	4	5	0	3
	Total positive features	195	202	263	269	177	184	185
	Frown brown	117	105	36	24	116	114	126
Loss	Closing eyes	39	49	57	63	62	57	45
	Head down	6	2	2	2	4	3	3
	Total negative features	162	156	95	89	182	174	174

## Data Availability

The data presented in this study are available on request from the corresponding author. The data are not publicly available due to privacy reasons.
